# Tuberculosis and other bacterial co-infection in Cambodia: a single center retrospective cross-sectional study

**DOI:** 10.1186/s12890-019-0828-4

**Published:** 2019-03-11

**Authors:** Engi F. Attia, Yaty Pho, Somary Nhem, Chandara Sok, Borady By, Dariven Phann, Huy Nob, Sovanndeth Thann, Sinath Yin, Rachael Noce, Chamrouensann Kim, Joanne Letchford, Thomas Fassier, Sarin Chan, T. Eoin West

**Affiliations:** 1 International Respiratory and Severe Illness Center, University of Washington, Seattle, WA USA; 2 Division of Pulmonary, Critical Care, and Sleep Medicine, Department of Medicine, University of Washington, 325 Ninth Avenue, Box 359640, Seattle, WA 98104 USA; 3 Kampong Cham Provincial Hospital, Kampong Cham, Cambodia; 4 University of Washington Medical Center and Department of Global Health, University of Washington, Seattle, WA USA; 5 Médecins Sans Frontières France, Kampong Cham, Cambodia; 6 Diagnostic Microbiology Development Program, Phnom Penh, Cambodia; 7 University of Health Sciences, Phnom Penh, Cambodia; 8 Calmette Hospital, Phnom Penh, Cambodia

**Keywords:** Lung, Pneumonia, Tuberculosis, Diagnostics, Respiratory infection, Cambodia

## Abstract

**Background:**

Cambodia, a lower middle-income country of about 16 million individuals in southeast Asia, endures a high burden of both tuberculosis and other lower respiratory infections. Differentiating tuberculosis from other causes of respiratory infection has important clinical implications yet may be challenging to accomplish in the absence of diagnostic microbiology facilities. Furthermore, co-infection of tuberculosis with other bacterial lower respiratory infections may occur. The objective of this study was to determine the prevalence and etiologies of tuberculosis and other bacterial co-infection and to analyze the clinical and radiographic characteristics of patients presenting with respiratory infection to a provincial referral hospital in Cambodia.

**Methods:**

We performed a retrospective, cross-sectional analysis of laboratory and clinical data, on patients presenting with respiratory symptoms to a chest clinic of a 260-bed provincial referral hospital in Cambodia. We analyzed mycobacterial and bacterial sputum test results, and demographics, medical history and chest radiography.

**Results:**

Among 137 patients whose treating clinicians ordered sputum testing for tuberculosis and other bacteria, the median age was 52 years, 54% were male, 3% had HIV infection, and 26% were current smokers. Nearly all had chronic respiratory symptoms (> 96%) and abnormal chest radiographs (87%). Sputum testing was positive for tuberculosis in 40 patients (30%) and for bacteria in 60 patients (44%); 13 had tuberculosis and bacterial co-infection (9% overall; 33% of tuberculosis patients). Clinical characteristics were generally similar across pulmonary infection types, although co-infection was identified in 43% of patients with one or more cavitary lesions on chest radiography. Among those with bacterial growth on sputum culture, Gram negative bacilli (*Klebsiella* and *Pseudomonas* spp.) were the most commonly isolated.

**Conclusions:**

Among patients with symptoms of respiratory infections whose treating clinicians ordered sputum testing for tuberculosis and other bacteria, 9% of all patients and 33% of tuberculosis patients had tuberculosis and bacterial co-infection. Greater availability of microbiologic diagnostics for pulmonary tuberculosis and bacterial infection is critical to ensure appropriate diagnosis and management.

## Background

Lower respiratory infections (LRIs) are a major cause of morbidity and mortality worldwide, especially in resource-limited settings such as Cambodia, a lower middle-income country of about 16 million individuals in southeast Asia [1]. While the lack of diagnostic microbiology facilities has limited study of LRI etiologies in Cambodia, available data suggest that *Streptococcus pneumoniae*, *Haemophilus influenzae*, *Pseudomonas aeruginosa*, *Klebsiella pneumoniae*, and *Burkholderia pseudomallei* are the most common etiologies of community-acquired pneumonia (CAP) in Cambodia and its neighbors [2]. *Mycobacterium tuberculosis* infection (TB) is also a major cause of respiratory disease in Cambodia, and the incidence of TB in 2017 (326 per 100,000 population) was among the highest in the world [3].

Co-infection with TB and bacterial pathogens has been described, particularly in populations with a high TB prevalence [4–8]. Differentiating TB from other LRIs such as bacterial pneumonia is an important clinical challenge in these settings, and inability to differentiate TB from other LRIs may result in poorer health outcomes [9–11]. In this study, we analyzed data from patients presenting with symptoms of respiratory infection at a Cambodian provincial hospital who had both mycobacterial and other bacterial testing performed to determine the prevalence and etiologies of bacterial co-infection in patients with tuberculosis.

## Methods

### Study population, design and site

We performed a retrospective, cross-sectional study at Kampong Cham Provincial Hospital (KCPH) in central Cambodia, analyzing data from November 2012 through December 2013. KCPH is a 260-bed government referral hospital serving a population of nearly 2 million people situated on the Mekong river, 127 km by road north-east of Cambodia’s capital Phnom Penh. We selected this hospital because of the TB clinical/diagnostic laboratory and diagnostic microbiology laboratory capacity supported by partners Médecins Sans Frontières France and the Diagnostic Microbiology Development Program. The hospital offers a Chest Clinic for initial triage and evaluation of patients presenting with symptoms of respiratory infection, as well as both comprehensive mycobacterial and other bacterial diagnostic capacity for sputum specimen analysis [12]. The evaluating physician determines whether patients require hospitalization or if outpatient management in the clinic is appropriate. Mycobacterial and other bacterial testing is ordered based on clinical suspicion and on local standards of care. Almost all adult sputum samples are spontaneously produced. Diagnostic testing is performed in a dedicated TB laboratory and in an adjacent bacterial microbiology laboratory. TB testing includes smear, culture using the BBL MGIT (Mycobacteria Growth Indicator Tube) Manual System (Becton Dickinson, Sparks, MD, USA) and/or Lowenstein-Jensen media, and Xpert MTB/RIF [Cepheid, Sunnyvale, CA, USA]). For bacterial testing, sputum specimens are evaluated first by Gram stain and acceptable samples are cultured onto sheep blood, chocolate, and MacConkey agars; bacteria are subsequently identified by standard laboratory procedures.

The inclusion criteria for this analysis were individuals with symptoms of respiratory infection presenting to the KCPH Chest Clinic who underwent mycobacterial and bacterial sputum testing within 14 days of each other, and who had a sputum sample deemed acceptable for bacterial culture (few [< 10] epithelial cells or moderate/high leukocytes). For this study, sputum positive for mycobacterial culture or Xpert assay was considered pulmonary TB; sputum acceptable for bacterial culture growing a potentially pathogenic (non-mycobacterial) bacterial organism was considered bacterial respiratory infection (as opposed to colonization).

### Data collection

We abstracted a) results of acid-fast bacilli (AFB) stain, mycobacterial culture, and Xpert MTB/RIF assays on sputum specimens from the TB laboratory records; b) Gram stain (number of squamous epithelial cells and leukocytes) and bacterial culture on sputum specimens from the bacterial microbiology laboratory records; and c) corresponding demographic information and clinical data such as medical history and clinician interpretations of chest radiographs from the Chest Clinic records.

### Data analysis

Categorical data are presented as number and proportions, and are compared using χ^2^ or Fisher’s exact tests. Continuous data are presented as median and interquartile range, and are compared using a one-way analysis of variance (ANOVA).

### Ethics

Human subjects approval was granted by the National Ethics Committee of Health Research of Cambodia and by the University of Washington Institutional Review Board. All procedures performed in studies involving human participants were in accordance with the ethical standards of the institutional and/or national research committee and with the 1964 Helsinki declaration and its later amendments or comparable ethical standards. For this type of study, formal consent is not required.

## Results

One hundred and thirty-seven patients underwent both mycobacterial and bacterial sputum testing within 14 days and had acceptable sputum samples for bacterial culture. The median age was 52 years and 54% were male (Table 1). Three percent had known HIV infection, 26% were current cigarette smokers, and 37% had previously received treatment for prior pulmonary TB. At the time of presentation, almost all (96%) had cough. Other common symptoms were weight loss (66%) and shortness of breath (54%). Hemoptysis was only reported by 20%. The median duration of dyspnea, cough, chest pain, fever and weight loss ranged from 30 to 60 days whereas the median duration of hemoptysis was 10 days (data not otherwise shown). Half (50%) reported taking antimicrobials prior to presentation. Chest radiographs at presentation were normal in only 13%; 10% had one or more cavitary lesions, 61% had infiltrates and/or consolidations, 12% had fibrosis, and only 3% had bronchiectasis or bronchitis.

**Table 1:** Characteristics of 137 individuals with mycobacterial and bacterial sputum sample tests

	Median (IQR) or N (%)
Age (yrs)	52 (37–64)
Male	74 (54)
HIV infection	4 (3)
Current smoking	35 (26)
Previous pulmonary TB	51 (37)
Number of times TB treated^a^	
1	41/50 (80)
2+	10/50 (20)
Symptoms at presentation	
Shortness of breath	74 (54)
Cough	132 (96)
Hemoptysis	28 (20)
Chest pain	68 (50)
Fever	42 (31)
Weight loss	90 (66)
Reported medication use at presentation	
Antimicrobials prior to presentation	68 (50)
Antibacterials	10/68 (15)
Anti-TB	2/68 (3)
Unknown	56/68 (82)
Other medications prior to presentation	71 (52)
Clinical chest x-ray interpretation	
Normal chest x-ray	18 (13)
Cavitary lesion(s)	14 (10)
Infiltrates and/or consolidation	84 (61)
Fibrosis	17 (12)
Bronchiectasis and/or bronchitis	4 (3)
Pathogenic organisms identified in sputum sample	
None	50 (37)
TB only	27 (20)
Bacteria only	47 (34)
TB and bacteria	13 (9)

^a^Prior TB treatment data were missing for one patient reporting previous pulmonary TB

Sputum mycobacterial testing was positive for TB in 40 patients (29%, Table 1); 38 patients (28%) had growth of *M. tuberculosis* on culture, and 26 (19%) had a positive Xpert test. None of the positive Xpert tests for *M. tuberculosis* was positive for rifampin resistance. Other (non-mycobacterial) bacteria were isolated from the sputum specimens of 60 patients (44%). Thirteen patients (9% of all patients; 33% of TB patients) had TB and bacterial co-infection. No potentially pathogenic bacteria or TB were identified in 50 patients (37%). Overall, clinical characteristics at presentation did not differ substantially between those with pulmonary TB, bacterial organisms, or TB and bacterial co-infection (Table 2). A notable exception was that, in contrast to patients with other sputum test results, the majority of patients with growth of bacterial organisms had previous pulmonary TB (*N* = 25/47, 53%).

**Table 2:** Characteristics of 137 individuals with mycobacterial and bacterial sputum sample tests stratified by test results

	No identified TB or bacteria (*N* = 50)	TB only (*N* = 27)	Bacteria only (*N* = 47)	TB and bacteria (*N* = 13)	*p*-value
Age (yrs)	55 (44–64)	42 (32–61)	53 (38–65)	52 (45–62)	0.10
Male	24 (48)	17 (63)	26 (55)	7 (54)	0.65
HIV infection	3 (6)	1 (4)	0	0	0.35
Current smoking	11 (22)	9 (33)	9 (19)	6 (46)	0.17
Previous pulmonary TB	20 (40)	3 (11)	25 (53)	3 (23)	0.002
Number of times TB treated^a^					0.10
1	18/20 (90)	1 (33)	20 (80)	2 (67)	
2+	2/20 (10)	2 (67)	5 (20)	1 (33)	
Symptoms at presentation					
Shortness of breath	29 (58)	19 (70)	21 (45)	5 (38)	0.11
Cough	50 (100)	26 (96)	43 (91)	13 (100)	0.13
Hemoptysis	6 (12)	4 (15)	15 (32)	3 (23)	0.09
Chest pain	25 (50)	14 (52)	21 (45)	8 (62)	0.76
Fever	12 (24)	13 (48)	13 (28)	4 (31)	0.17
Weight loss	32 (64)	20 (74)	31 (66)	7 (54)	0.64
Reported medication use at presentation					
Antimicrobials prior to pesentation	25 (50)	18 (67)	19 (40)	6 (46)	0.18
Antibacterials	2/25 (8)	4/18 (22)	3/19 (16)	1/6 (17)	–
Anti-TB	0/25 (0)	2/18 (11)	0/19 (0)	0/6 (0)	–
Unknown	23/25 (92)	12/18 (67)	16/19 (84)	5/6 (83)	–
Other medications prior to presentation	28 (56)	16 (59)	22 (47)	5 (38)	0.51

NOTE: Data reported as median (IQR) or N (%)

^a^Prior TB treatment data were missing for one patient reporting previous pulmonary TB

Among the sixty patients with bacterial growth on sputum culture, Gram negative bacilli were the most commonly isolated organisms (Table 3). *Klebsiella* spp. were the most common (*N* = 28, 20%) followed by *Pseudomonas* spp. (*N* = 20, 15%). The distribution of bacterial organisms did not differ substantially between those with and without TB co-infection.

**Table 3:** Bacterial sputum culture results^a^

	Entire cohort *N* = 137	Bacteria only *N* = 47	TB and bacteria *N* = 13
Normal flora	77 (56)	0	0
*Klebsiella* spp.	28 (20)	22 (47)	6 (46)
*Pseudomonas* spp.	20 (15)	17 (36)	3 (23)
*Escherichia coli*	3 (2)	2 (4)	1 (8)
*Enterobacter* spp.	3 (2)	3 (6)	0
*Staphylococcus aureus*	3 (2)	3 (6)	0
*Haemophilus influenzae*	2 (2)	1 (2)	1 (8)
*Acinetobacter baumanii*	2 (2)	0	2 (15)
*Burkholderia pseudomallei*	1 (1)	1 (2)	0
*Burkholderia cepacia*	1 (1)	1 (2)	0
*Stenotrophomonas maltophila*	1 (1)	1 (2)	0
Other Gram negative bacilli	1 (1)	1 (2)	0

^a^Percents add to > 100% because 5 cultures grew > 1 organism:

1 grew *B. pseudomallei* + *Klebsiella* spp.

3 grew *Klebsiella* spp. + *Pseudomonas* spp.

1 grew *Pseudomonas* spp. + *S. aureus*

**Fig. 1:**
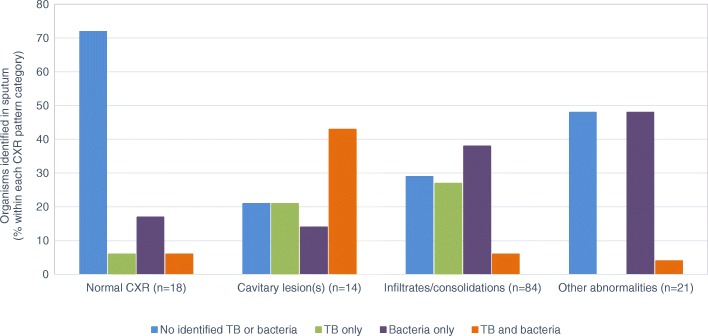
Organisms identified in sputum stratified by chest radiograph findings. The exact test *p*-value comparing types of identified organisms in the sputum (TB and/or bacteria) across categories of chest radiograph findings was < 0.001. The “Other abnormalities” category comprises fibrosis, bronchiectasis, and bronchitis

One hundred and nineteen patients (87%) had chest radiographs that were considered abnormal by treating clinicians. The distribution of organisms identified in sputum differed across categories of chest radiographs (*p* < 0.001). Among the subset of patients with normal chest radiographs, neither bacteria nor TB were identified in the majority (13/18, 72%) (Fig. 1). Presence of infiltrates and/or consolidations was the most common radiographic abnormality among all patients (84/137, 61%). Of individuals with this radiographic pattern, similar numbers had either no identified infectious etiology (24/84, 29%), bacterial infection (32/84, 38%) or TB (23/84, 27%). However, fewer had TB and bacterial co-infection (*N* = 5, 6%). In contrast, among patients with cavitary lesions on chest radiographs, TB and bacterial co-infection (6/14, 43%) was the most common microbiological result.

## Discussion

The main finding of this study is that 9% of all patients and 33% of TB patients evaluated for respiratory symptoms who underwent both mycobacterial and bacterial sputum testing at a Cambodian provincial hospital had TB and (non-mycobacterial) bacterial co-infection detected. Moreover, in this population, *Klebsiella* and *Pseudomonas* were the dominant causes of respiratory infection with and without TB co-infection.

Without the appropriate diagnostic modalities, the presence of either or both TB and bacterial respiratory infection is often difficult to distinguish. Our findings suggest that TB and bacterial co-infection may be more likely among patients with cavitary lesions on chest radiography. Another study of patients with acute LRI in Cambodian provincial hospitals found that Gram negative bacteria were more likely to be cultured from patients whose chest radiographs had pulmonary sequelae of prior infections, including TB, compared to those whose chest radiographs were normal [13]. Nonetheless, as in other studies, [5, 6, 13] our results highlight that clinical and radiographic characteristics are insufficient to meaningfully distinguish between pulmonary TB, bacterial infection and TB/bacterial co-infection in TB endemic regions, given overlapping presentations.

Few published data describe the burden of co-infection with TB and other pathogens in the general populations of regions where TB prevalence is high and TB treatment is commonly based on presumptive diagnoses [5–8]. For instance, in Kenya, ~ 8% of patients hospitalized with CAP had TB and bacterial co-infection [8]. In the same Cambodian hospital as our study, nearly 11% of patients with suspected pulmonary TB had growth of non-tuberculous mycobacterial isolates on sputum culture, and 4 of 217 patients with pulmonary TB were also diagnosed with non-tuberculous mycobacterial pulmonary disease [12]. Our study provides further evidence that co-infection of other respiratory pathogens with TB is readily quantifiable, although the high rate of co-infection observed in TB-infected individuals (33%) may overestimate the true prevalence in all TB patients at this hospital. We only included patients in this study with sputum specimens that were tested for both mycobacterial and bacterial pathogens; yet, for the majority of patients presenting to the hospital with respiratory symptoms sputum samples are tested for TB alone. The patients we studied may therefore have been more likely to have had sequelae related to prior TB, non-resolving infection, immune compromise, or bronchiectasis.

Over half of the patients in our study were taking antimicrobials and other medications prior to presentation, raising concern that culture results may have been falsely negative or that underlying chronic comorbidities may play a role in LRI in resource-limited settings as has been found in higher resource countries [14]. It remains unknown whether pulmonary TB infection increases the risk of bacterial super-infection or whether acute presentation of pulmonary TB is precipitated by development of bacterial pneumonia. Regardless, misdiagnosis of either TB or other bacterial pulmonary infection has the potential to lead to poorer outcomes, including increased healthcare costs, antimicrobial resistance and mortality [9–11].

Our finding that Gram negative bacteria, including *Klebsiella* and *Pseudomonas,* are the most common pathogens identified among the patients in this study is consistent with published data highlighting the incidence and prevalence of bacterial CAP in Cambodia and neighboring countries [2, 15]. Furthermore, we did not identify any cases of *S. pneumoniae* infection in this study. The lack of *S. pneumoniae* and the relative abundance of Gram negative bacteria cultured from respiratory specimens may be due to antibiotic pre-treatment in our population. Gram negative bacteria cultured from sputum may also reflect colonization of abnormal lung architecture following previous TB or respiratory infection [16, 17]. Alternatively, this may represent a troubling epidemiologic transition of sorts – the review by Goyet et al. found that *S. pneumoniae* was frequently identified in earlier studies, but Gram negative bacteria were consistently identified more frequently among adults hospitalized with bacterial CAP during more recent studies [2]. Alarmingly, this trend may not be isolated to southeast Asia. Gram negative bacteria were isolated from nearly half of Nigerian TB patients receiving anti-TB therapy who presented with acute LRI [6].

An important strength of this study was our access to advanced, high-quality TB and microbiologic diagnostic data from well-established laboratories. Furthermore, we were able to link these data to clinical information, including clinical chest radiography. Our study also had several limitations. This was a single center, retrospective analysis with a small sample, limiting power to make statistical inferences. Our analysis was restricted to the subset of patients for whom both mycobacterial and bacterial testing was ordered by treating clinicians. Our results may not be globally generalizable, but, importantly, studies from the same region have identified similar bacterial pathogens in CAP. Samples were not analyzed for viral pathogens, which may have accounted for some instances in which chest radiography was abnormal but neither bacterial nor mycobacterial pathogens were identified. Additionally, positive bacterial sputum cultures could well represent colonization rather than true infection, especially among those with evidence of underlying chronic lung disease. We also could not distinguish between bronchiectasis exacerbations and pneumonia. HIV infection is a major risk factor for TB and HIV co-infection alters the presentation and outcome of TB, yet HIV infection in our study may have been under-reported. We did not have access to details of clinical treatment outcomes. Further, prospective studies are needed to address these limitations.

## Conclusions

Among patients with presumptive LRI who underwent mycobacterial and bacterial sputum testing, 9% of all patients and 33% of TB patients had TB and other bacterial co-infection. *Klebsiella* and *Pseudomonas* were the dominant bacterial pathogens cultured regardless of TB co-infection. Increasing the availability of resources for microbiologic diagnostics for pulmonary TB and bacterial pneumonia is critical to ensure appropriate administration of antimicrobial agents in this era of expanding antimicrobial resistance among mycobacterial and bacterial pathogens.

## Abbreviations

AFB: Acid-fast bacilli
ANOVA: One-way analysis of variance
CAP: Community-acquired pneumonia
KCPH: Kampong Cham Provincial Hospital
LRI: Lower respiratory infection
MGIT: Mycobacteria Growth Indicator Tube
TB: *Mycobacterium tuberculosis* infection/tuberculosis
